# Yeasts and Bacterial Consortia from Kefir Grains Are Effective Biocontrol Agents of Postharvest Diseases of Fruits

**DOI:** 10.3390/microorganisms8030428

**Published:** 2020-03-18

**Authors:** V. Yeka Zhimo, Antonio Biasi, Ajay Kumar, Oleg Feygenberg, Shoshana Salim, Silvana Vero, Michael Wisniewski, Samir Droby

**Affiliations:** 1 Department of Postharvest Science, Agricultural Research Organization (ARO), The Volcani Center, Rishon LeZion 7505101, Israel; 2 Área Microbiología, Departamento de Biociencias, Facultad de Química, Universidad de la República, Gral Flores 2124, Montevideo 11800, Uruguay; 3 Appalachian Fruit Research Station, Agricultural Research Service, United States Department of Agriculture, Wiltshire Road, Kearneysville, WV 25443, USA

**Keywords:** yeasts, bacteria, kefir, biological control, postharvest, *Penicillium*

## Abstract

Fungal pathogens in fruits and vegetables cause significant losses during handling, transportation, and storage. Biological control with microbial antagonists replacing the use of chemical fungicides is a major approach in postharvest disease control, and several products based on single antagonists have been developed but have limitations related to reduced and inconsistent performance under commercial conditions. One possible approach to enhance the biocontrol efficacy is to broaden the spectrum of the antagonistic action by employing compatible microbial consortia. Here, we explore commercial kefir grains, a natural probiotic microbial consortium, by culture-dependent and metagenomic approaches and observed a rich diversity of co-existing yeasts and bacterial population. We report effective inhibition of the postharvest pathogen *Penicillium expansum* on apple by using the grains in its fresh commercial and milk-activated forms. We observed few candidate bacteria and yeasts from the kefir grains that grew together over successive enrichment cycles, and these mixed fermentation cultures showed enhanced biocontrol activities as compared to the fresh commercial or milk-activated grains. We also report several individual species of bacteria and yeasts with biocontrol activities against *Penicillium* rots on apple and grapefruit. These species with antagonistic properties could be further exploited to develop a synthetic consortium to achieve enhanced antagonistic effects against a wide range of postharvest pathogens.

## 1. Introduction

Fungal pathogens are the main cause of postharvest losses in fruits and vegetables during storage, shipment, and post-consumer purchase [1,2]. In most crops, the pre and/or postharvest applications of synthetic fungicides have been the main approach used to control decay [3]. Nevertheless, chemical-based control approaches have been under mounting scrutiny by the public and regulatory authorities due to their negative effects on human and animal health, as well as the environment in general [3]. This concern has been the main driving force of research to identify effective and more eco-friendly methods for the management of postharvest fungal diseases [1,4].

Microbial biocontrol agents that inhibit the establishment and growth of postharvest pathogens have great potential as safe and effective alternatives to the use of synthetic fungicides [5,6,7]. The discovery and development of these biocontrol agents have been based on the paradigm of isolating a single antagonist that is effective against several different postharvest pathogens and was expected to be effective on different commodities that vary in their genetic background, physiology, pathogen susceptibility, and pre- and postharvest management practices. This paradigm has resulted in several limitations, including inconsistent efficacy and a narrow range of biocontrol activity on specific hosts or pathogens. These shortcomings are the reasons why the commercialization of postharvest biocontrol agents has had limited success [5]. Several commercial biological control products based on single antagonists have been developed, including Biosave™ (*Pseudomonas syringae* Van Hall) Aspire™ (*Candida oleophila*), Yieldplus™ (*Cryptococcus albidus*), ShemerTM (*Metschnikowia fructicola*), CandifruitTM (*Candida sake*), NexyTM, (*Candida oleophila*), and BoniProtectTM (*Aureobasidium pullulans*) [5,6]. A comprehensive discussion of the reasons these products have had limited commercial success was presented by Droby et al. [6], who highlighted the shortcomings related to reduced and inconsistent performance when biocontrol agents (BCAs) are used under commercial conditions. Several approaches have been suggested for improving the biocontrol efficacy of postharvest biocontrol agents. Attempts to enhance efficacy have included the use of combinations of two or more postharvest treatments, such as combining a BCA treatment with physical means (heat, hot water brushing), natural and food-grade chemicals, and different packaging techniques [4,7,8,9]. A unique approach to enhance the biocontrol efficacy that provides a new outlook to postharvest biocontrol is to broaden the spectrum of action of BCAs by utilizing compatible microbial consortia instead of single antagonists. Microbial consortia could comprise natural or synthetic mixtures of interacting microbial populations that thrive in many diverse environmental niches. The use of a consortium in biocontrol systems would have several advantages over the use of a single species, including the potential for a wider range of biocontrol efficacy, robustness, resilience to environmental stress, and modularity [10].

Fermented food products, including the communities of organisms responsible for their fermentation, have been consumed for centuries [11]. These communities of fermentative organisms are now recognized as probiotics that confer several health benefits to humans and animals [12]. Members of commensal microbiota occur naturally in fermented food products such as yogurt, kefir, sauerkraut, cabbage kimchee, and soybean-based miso and natto [13]. Kefir is a traditional beverage considered to be a nutritious “functional food” due to its health benefits [14]. It is produced by the fermentation of milk by microbial species present in “kefir grains” used as a starter culture [14]. Due to its microbial composition, kefir is considered to be a good source of probiotic microorganisms that have a positive effect on the human digestive system [15]. Kefir grains contain a natural microbial consortium composed of a diverse and stable community of bacterial and yeast species [15,16] embedded in an exopolysaccharide matrix called kefiran [17]. Several studies have demonstrated that the microbes present in kefir are probiotic have beneficial health properties, including antibiosis [18], hypocholesterolaemic [19], antihypertensive [20], and anti-inflammatory [21] effects, as well as antioxidant [22], and anti-carcinogenic activity [23].

The objective of the present study is to determine the composition of the microbiota in kefir grains and evaluate their biocontrol activity against postharvest pathogens. We assumed that the kefir grains comprise natural probiotic microbial consortia consisting of bacteria and yeasts with unique interactions and that these consortia can be used as a microbial-community-based biocontrol entity. Microbial diversity and abundance in different sources of kefir grains were characterized using a metagenomics approach. Classical culturing techniques were also employed to isolate and identify bacteria and yeast communities and test their biocontrol efficacy. We report on the isolation and identification of several yeast and bacterial genera that coexist in kefir grains and demonstrate that several of the isolated yeast and bacterial strains exhibit biocontrol potential.

## 2. Materials and Methods

### 2.1. Fungal Cultures and Fruit

*Penicillium expansum* and *P. digitatum* were isolated from decayed apple or grapefruit, respectively, and short-term cultures for use in biocontrol assays were maintained on potato dextrose agar (PDA; Difco, MD, USA). Spore suspensions of the pathogens were made in glycerol (30%) and kept at −80 °C for long term storage of the cultures, and were used to start new cultures every few weeks. Spore suspensions used in the biocontrol tests were obtained from two-week-old PDA cultures grown at 25 °C. Spore concentration was determined using a hemocytometer chamber (Neubauer Improved, Germany), and the final concentration was adjusted to 10^5^ conidia ml^−1^ with sterile distilled water. Apples (*Malus pumila*, cv. Granny Smith) and grapefruits (*Citrus* x *paradisi*, cv. Star Ruby) were harvested at commercial maturity and used immediately or stored at 2 or 8 °C, respectively, for later use. Fruit were washed with tap water before use, air dried, and the fruit surface was wiped with 70% ethanol.

### 2.2. Kefir Grains and Culture Conditions

Kefir grains from four commercial companies—Fusion Tea (Fusion Teas, USA), Starter Culture (Natural Probiotic Selection Ltd., UK), Florida Sun (Florida Sun Kefir, USA), and Body Ecology (Body Ecology Inc., USA)—were purchased through Amazon (amazon.com) and used in the present study. To obtain milk-activated (MA) kefir grains, the commercially-purchased grains were added to pasteurized full-fat, sheep milk (2 g in 200 mL) in 500 mL Erlenmeyer flasks and incubated, without shaking, at 20 °C for 24 h. The kefir grains were recovered from the milk cultures by passing the culture liquid through sterile gauze. The collected kefir grains were washed twice with sterile water to remove residual milk and again placed into a new milk culture. This was done for two additional cycles of incubation of 24 h. The MA-kefir grains obtained after the 3rd cycle were recovered and washed as described, air dried, and stored at −18 °C until use.

MA-kefir grains were subjected to several cycles of growth in liquid enrichment cultures to study the growth dynamics of the microbiota and to determine the predominant microbial communities that co-grow during the enrichment process. For that purpose, 1 g fw^−1^ of each of the MA grains was inoculated in 50 mL of de Man, Rogosa, and Sharpe (MRS) broth (Sigma-Aldrich, USA) supplemented with 1% (*w/v*) glucose in 150 mL Erlenmeyer flasks and incubated for 24 h at 28 °C on a rotary shaker incubator at 200 rpm. Subsequently, 1 mL aliquots were aseptically withdrawn from each culture and designated as the 1st cycle of enrichment and transferred to fresh medium and grown at 24 h for a 2nd cycle. This procedure was repeated additional times to have, at the end, a total of 5 cycles of enrichment.

### 2.3. Culture-Dependent Microbial Characterization, Community Dynamics, and Diversity Analysis of Kefir Grains

Culturable yeasts and bacteria and their population dynamics in kefir grains were determined from each of the commercial products, as well as MA-kefir grains, and from each of the enrichment cycles using serial dilutions in saline (0.9% NaCl) that were plated on Luria–Bertani agar (LB, Difco, MD, USA) and yeast extract peptone dextrose agar (YPD; Difco, MD, USA) Petri plates to obtain total bacterial and yeast counts, respectively. Dilutions from the commercial and MA-kefir grains were prepared following homogenizing of 1 g (fw, fresh weight) of each sample in sterile saline water. Dilutions from the enrichment cultures were made following precipitation of the cells by centrifugation (10,000× *g*, 5 min, 20 °C), and washing of the obtained pellets with sterile saline and resuspension to their initial volume. After 3–4 days of incubation at 28 °C, viable cell counts were determined and the results expressed as means of three replications each of colony-forming units (CFU) per gram (grains) or per ml (enrichment culture media) of sample ± standard deviations.

For the analysis of species diversity, bacterial and yeast colonies were randomly picked (10% to 20% of the total colony count) from the agar plates (total of 382 colonies) and streaked on fresh LB or YPD plates to obtain pure cultures. Selected bacterial and yeast cultures were stored at −80 °C in 30% glycerol for later use. Bacterial and yeast DNA from the cultures was extracted and purified using a Wizard Genomic DNA Purification Kit (Promega, Madison, WI, USA), following the manufacturer’s instructions. The genomic DNA of the selected isolates was subjected to a PCR assay to amplify the 16S rRNA gene (approximately 300 bp) for the bacterial isolates with primer pair 515F (5′-GTGCCAGCMGCCGCGGTAA-3′) and 806R (5′ -GGACTACHVGGGTWTCTAAT-3′) [24]. The primer pair used for the yeast isolates to amplify the D1–D2 region (approximately 600 bp) in the 25S rRNA gene was NL1 (5′-GCATATCAATAAGCGGAGGAAAAG-3′) and NL4 (5′-GGTCCGTGTTTCAAGACGG-3) [25]. PCR products were sequenced and taxa determined by identifying the highest sequence homology using the BLAST algorithm and the GenBank database (http://blast.ncbi.nlm.nih.gov/).

### 2.4. Genomic DNA Isolation from Kefir Grains

Fresh and MA-kefir grain samples (1 g fw) were homogenized in 10 mL 0.9 % NaCl (*w/v*). Then 2 mL of the homogenate was centrifuged (10,000× *g*, 5 min, 20 °C) and the obtained pellet was washed twice with sterile distilled water. Two ml of the suspensions of microbial cells from each of the five cycles of enrichment cultures were pelleted and washed twice with sterile distilled water. Total genomic DNA was extracted from the pellets using a Wizard Genomic DNA Purification kit (Promega, Madison, WI, USA) following the manufacturer’s instructions. The extracted DNA was stored at –20 °C.

### 2.5. Library Preparation, Metagenomic Sequencing, and Bioinformatics

The quantity of each of the DNA samples was determined using a spectrophotometer (Nanodrop; Thermo Fisher Scientific Inc.), and the total DNA concentration was adjusted to 5.0 ng μL^−1^. The fungal ITS2 region was amplified using the universal primers ITS3/KYO2 and ITS4 to amplify the ITS2 region of ribosomal DNA [26]. The bacterial 16S region was amplified using the protocol described by Lundberg *et al* [27]. The universal 16S primer pair 515F and 806R was used to generate 16S amplicons [27]. All primers were modified to include Illumina adaptors (www.illumina.com). PCR reactions were conducted in a total volume of 25 μL containing 12.5 μL of KAPA HiFi HotStart ReadyMix (Kapa Biosystems, Wilmington, MA, USA), 1.0 μL of each primer (10 μM), 2.5 μL of DNA template, and 8.0 μL nuclease-free water. The amplifications were conducted in a T100 thermal cycler (Bio-Rad) using the following protocol: 3 min at 98 °C followed by 30 cycles of 30 s at 95 °C, 30 s at 50 °C, and 30 s at 72 °C. All amplifications ended with a final extension of 1 min at 72 °C. Nuclease free water (QIAGEN, Valencia, CA, USA) replaced template DNA in negative controls. All amplicons and amplification mixtures, including negative controls, were sequenced on a MiSeq platform using V2 chemistry (Illumina, San Diego, CA, USA). Illumina adaptors were then clipped and low-quality reads were removed by Trimmomatic 0.36 [28]. Paired-end reads were merged utilizing PEAR [29] for the 16S rRNA gene region and PANDAseq [30] for ITS rRNA gene region sequences with default parameters. Chimeric sequences were identified and removed using USEARCH [31] and VSEARCH 1.4.0 [32] for the 16S rRNA gene and ITS rRNA gene region sequences, respectively. UCLUST algorithm [31], as implemented in QIIME 1.9.1 [33], was used to cluster sequences queried against the Greengenes 13_8_97 database for 16S rRNA genes [34] and the UNITE dynamic database released on 01.12.2017 [35] at a similarity threshold of 97% for ITS genes. Sequences that failed to cluster against the database were de novo clustered using the same algorithm. After removing singletons, the most abundant sequences in each OTU were selected as representative sequences and used for the taxonomic assignment using the BLAST algorithm [36,37], as implemented in QIIME 1.9.1.

### 2.6. Biocontrol Activity of Kefir Grains, and Enrichment Cultures on Apples

Biocontrol potential of commercial, MA-kefir grains, and enrichment culture suspensions derived from the kefir grains were tested against *P. expansum* on “Granny Smith” apples. Two wounds (1 × 2 mm) were made with a sterile nail on the equator of each apple fruit. Suspensions of commercial and MA-kefir grains were prepared by homogenizing 1 g of grains in 1 mL of sterile distilled water, and then 1:10 dilutions were prepared. One ml aliquots directly drawn from each of the enrichment cycles were used in all the tests after pelleting the cells by centrifugation (10,000× *g*, 5 min, 20 °C), washing three times to remove any remaining growth medium and re-suspending the pellets in sterile distilled water to their initial volume. Then, 20 μL of the test suspension was administered into each wound. The wounds were allowed to dry and then inoculated with 30 μL of *P. expansum* spores (10^5^ spores/mL) or 30 μL sterile distilled water as a control. Each treatment was replicated three times, and each replicate consisted of 5 fruits with two wounds each. Treated fruit were placed in plastic trays and enclosed in polyethylene bags to maintain high humidity. Percent infected wounds and lesion diameter of the infected wounds were determined at 3, 4, 5, 6, and 7 days of storage at 20 °C.

### 2.7. Biocontrol Activity of Yeasts and Bacteria Isolated from Kefir Grains

Select bacteria and yeast isolates obtained from kefir grains and enrichment cultures were individually grown in 100 mL of either LB or YEPD (Difco, MD, USA) for 24 h at 28 °C on an orbital shaker set at 200 rpm. Cells were pelleted by centrifugation (10,000× *g*, 5 min, 20 °C), washed three times in sterile distilled water, re-suspended to their initial volume, and the cell concentration was adjusted as required. Biocontrol efficacy was tested on harvested apple (“Granny Smith”) and grapefruit (“Star Ruby”) against *P. expansum* and *P. digitatum*, respectively, as previously described.

## 3. Results

### 3.1. Microbial Composition and Diversity of Fresh and Milk-Activated (MA) Kefir Grains

**Figure 1:**
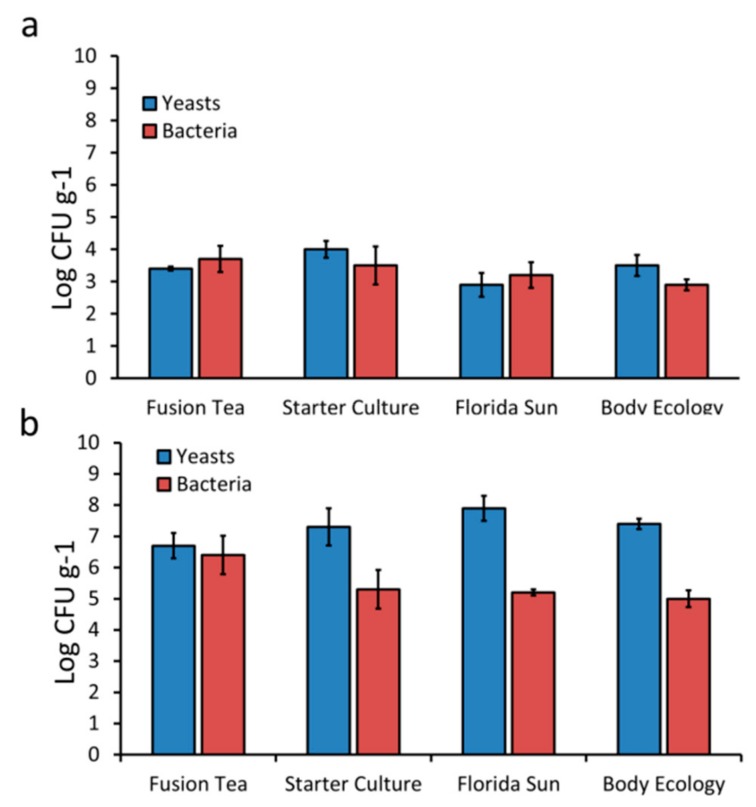
Total cultivable counts of yeasts and bacteria from fresh (**a**) and milk activated (**b**) kefir grains. Bars indicate standard errors of the means.

The microbial composition and level of diversity of kefir grains obtained from different commercial sources were determined using both culture-dependent and metagenomic approaches. The total viable counts of yeasts and bacteria in kefir grain samples are presented in Figure 1. The total viable counts of yeasts in fresh grains ranged from 1.3 × 10^3^ CFU g^−1^ in Florida Sun to 1.4 × 10^4^ CFU g^−1^ in Starter Culture. Aerobic bacteria counts were lowest in body ecology (1.3 × 10^3^ CFU g^−1^) and highest in Florida Sun (7.5 × 10^3^ CFU g^−1^). In contrast, MA-kefir grains had higher ranges of viable counts for both yeasts (1.4 × 10^7^ CFU g^−1^ in Fusion Tea to 1.3 × 10^8^ CFU g^−1^ in Florida Sun) and aerobic bacteria (1.4 × 10^5^ CFU g^−1^ in Body Ecology to 1.2 × 10^7^ CFU g^−1^ in Fusion Tea). Yeast cell counts were higher than bacterial cell counts in both fresh and MA-kefir grains, regardless of the commercial source.

**Figure 2:**
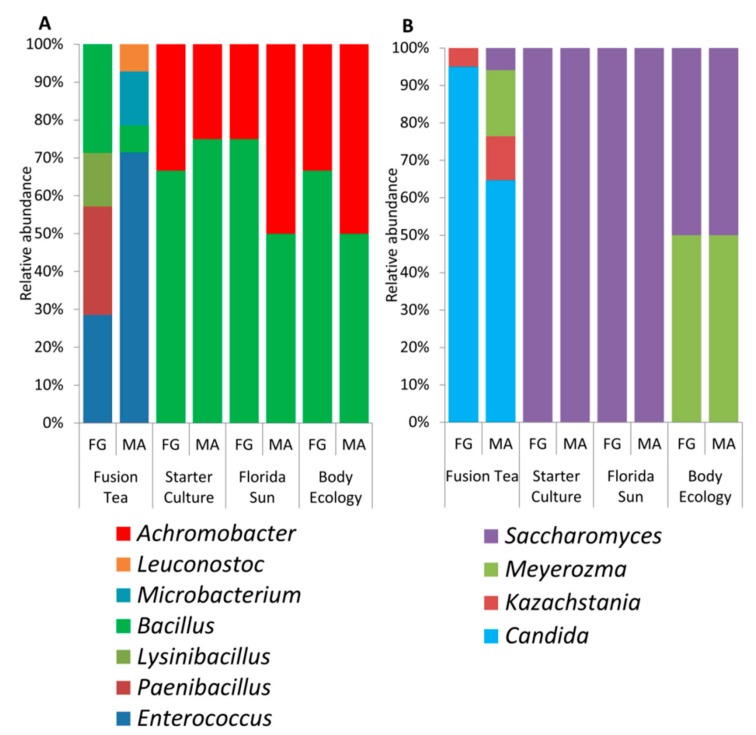
Diversity and relative abundance of bacterial (**a**) and yeast genera (**b**) isolated by culture-dependent approach from fresh (FG) and milk-activated (MA) grains.

Yeast and bacterial cultures (200 yeasts and 182 bacteria) isolated from all of the commercial samples of kefir grains were identified by partial ITS or 16S rRNA gene sequencing. Results revealed the presence of a total of 7 bacterial genera (*Achromobacter*, *Leuconostoc*, *Bacillus*, *Paenibacillus*, *Lysinibacillus*, *Enterococcus* and *Microbacterium*) and 4 yeast genera (*Saccharomyces*, *Meyerozyma*, *Candida*, and *Kazachstania*). Their relative abundances are presented in Figure 2. Among the products, Fusion Tea had the highest diversity of microbes isolated (4 yeasts and 6 bacterial genera) followed by Body Ecology (2 yeasts and 2 bacterial genera) while all bacteria obtained from Starter Culture and Florida Sun fell under 2 bacterial genera (*Bacillus* and *Achromobacter*) and all the yeasts belonged to *Saccharomyces* genera. *Bacillus* and *Saccharomyces* were the common genera identified from all the commercial sources of kefir grains. Notably, *Candida* and *Kazachstania* were unique to kefir grains in the Fusion Tea product.

**Figure 3:**
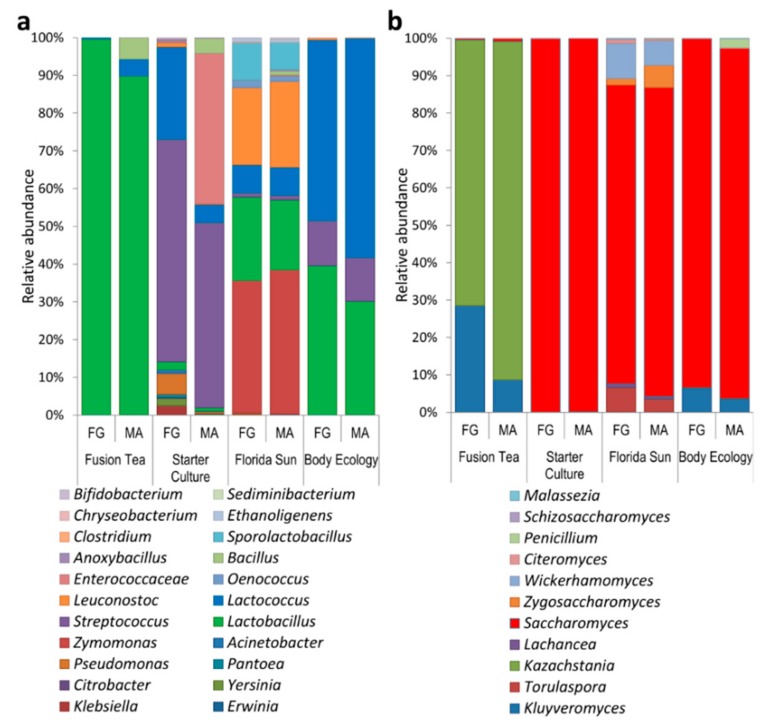
Diversity and relative abundance of dominant bacteria (**a**) and yeast (**b**) genera as identified by metagenomic approach from fresh (FG) and milk-activated (MA) grains.

Metagenomic analysis of the kefir grain products (fresh and MA) revealed a higher level of microbial diversity compared to the culture-dependent approach. The 16S reads from all the product samples combined were assigned to 44 genera. The relative abundance and distribution of the most abundant bacterial genera are presented in Figure 3a. The most dominant genera, comprising 90% of the identified genera in both fresh and MA-kefir grains, were *Lactobacillus*, *Lactococcus,* and *Bacillus* (in decreasing order of abundances) in the Fusion Tea product; *Streptococcus*, *Lactococcus,* and *Enterococcus* in the Starter Culture product; *Zymomonas*, *Lactobacillus*, *Leuconostoc,* and *Sporolactobacillus* in the Florida Sun product, and *Lactobacillus*, *Lactococcus,* and *Streptococcus* in the Body Ecology product. The genera, *Aeromonas*, *Carnobacteriaceae*, *Macrococcus*, *Brochothrix*, *Exiguobacterium*, *Chryseobacterium*, *Wautersiella*, *Myroides*, *Arthrobacter,* and *Pseudoclavibacter* were unique to the Starter Culture product. *Reyranella*, *Mesorhizobium*, *Achromobacter*, *Ralstonia*, *Variovorax*, *Ethanoligenens*, *Alistipes,* and *Sediminibacterium* were unique to the Florida Sun product and *Stenotrophomonas* was unique to the Fusion Tea product.

The ITS reads were assigned to 46 genera, and the relative abundance and distribution of the most abundant fungal genera are presented in Figure 3b. The most abundant genera in the fusion Tea product were *Kazachstania* (90.4% in MA-kefir, 71.0% in fresh grains) and *Kluyveromyces* (8.6% in MA-kefir, 28.5% in fresh and grains). The predominant yeast genus in Starter Culture product was *Saccharomyces* (99.7% in MA-kefir, 99.8% in fresh grains), while the most abundant genera in the Florida Sun product were *Saccharomyces* (82.4% in MA-kefir and 79.7% in fresh grains), and *Wickerhamomyces* (6.6% in MA-kefir, 9.54% in fresh grains). In the Body Ecology product, *Saccharomyces* was predominant (93.0% in MA, 92.8% in fresh grains) followed by *Kluyveromyces* (3.5% in MA, 6.4% in fresh). The genera *Talaromyces*, *Sarcinomyces*, *Acremonium*, *Rhodosporidium*, *Quambalaria*, *Sympodiomycopsis*, *Tilletiopsis*, *Ustilago,* and *Wallemia* were unique to the Body Ecology product, *Udeniomyces*, *Itersonilia,* and *Mrakiella* were unique to the Starter Culture product, while *Exophiala*, *Knufia*, *Bullera*, *Bensingtonia,* and *Erythrobasidium* were unique to the Florida Sun product.

### 3.2. Microbial Dynamics in Successive Enrichment of Kefir Grain Cultures

**Figure 4:**
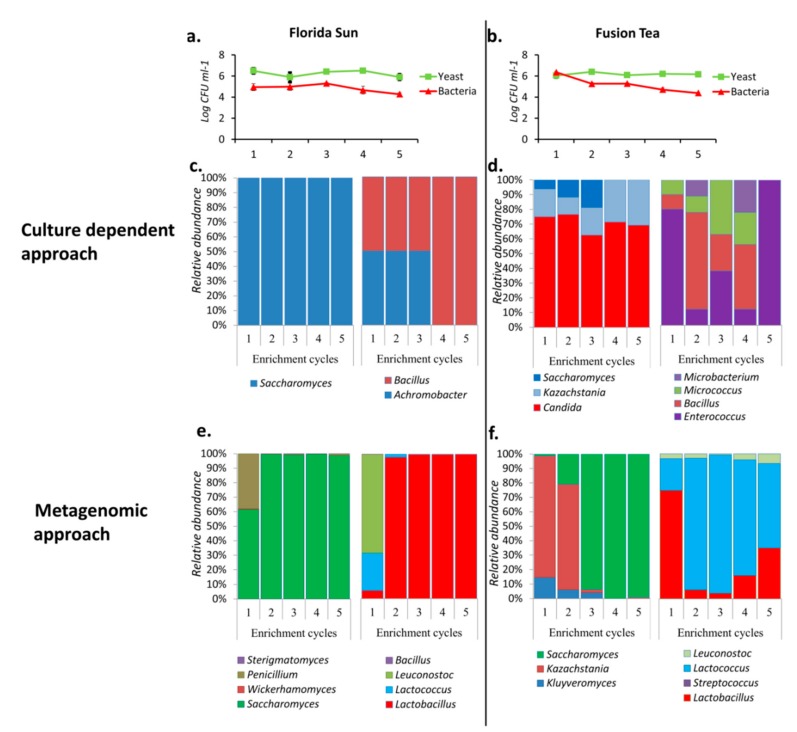
Population dynamics (CFUs) of yeasts and bacteria in Florida Sun (**a**) and Fusion Tea (**b**) and relative abundances and dynamics of the dominant yeast and bacterial taxa during five successive enrichment cycles of growth of kefir grains obtained by culture dependent (**c**—Florida Sun; **d**—Fusion Tea) and metagenomics approach (**e**—Florida Sun; **f**—Fusion Tea).

Florida Sun and Fusion Tea kefir grains displayed good biocontrol activity in initial experiments, and so were further studied to better understand the growth dynamics of the microbial communities present in these grains. Each of MA-kefir grains from these two products was passed through a series of five 24 h cycles of growth in enrichment cultures. This protocol was used to determine which microorganism(s) continue to dominate in the cultures through successive cycles of enrichment. The yeast population in the cultures derived from the Florida Sun product remained stable through all of the enrichment cycles, starting at 4.6 × 10^6^ CFU ml^−1^ in the 1st cycle and ending at 1.2 × 10^6^ CFU ml^−1^ in the 5th cycle. The bacterial population decreased marginally from 1.4 × 10^5^ CFU ml^−1^ in the 1st cycle to 2.3 × 10^5^ CFU ml^−1^ in the last enrichment cycle (Figure 4a). Similarly, the yeast population in the cultures derived from the Fusion Tea grains maintained a stable yeast population, beginning at 1.5 × 10^6^ CFU ml^−1^ in the 1st cycle and ending at 1.2 × 10^5^ CFU ml^−1^ in the 5th cycle, while the bacterial count decreased from 2.4 × 10^6^ CFU ml^−1^ in the 1st cycle to 2.5 × 10^4^ CFU ml^−1^ in the 5th cycle (Figure 4b).

The population dynamics in the enrichment cultures were also determined by using the culture-dependent approach. All the yeast isolates obtained from Florida Sun enrichment cultures were identified as belonging to the genus *Saccharomyces,* while all the bacteria isolated belonged to *Bacillus* and *Achromobacter* until the 3rd enrichment cycle, after which only *Achromobacter* was identified in the 4th and 5th cycles (Figure 4c). In contrast, yeasts isolated from Fusion Tea enrichment cultures belonged to 3 genera, namely, *Saccharomyces*, *Kazachstania,* and *Candida,* during the first three cycles, after which only *Kazachstania* and *Candida* were identified during the 4th and 5th cycles. Also, four bacterial genera, namely, *Enterococcus*, *Bacillus*, *Micrococus* and *Microbacterium,* were identified in varying proportions during the first four enrichment cycles, while only *Enterococcus* sp. was observed in the 5th enrichment cycle of Fusion Tea product (Figure 4d).

Analysis of the data obtained using the metagenomic approach resulted in the identification of four genera of fungi, *Saccharomyces*, *Wickerhamomyces*, *Penicillium,* and *Sterigmatomyces* in all of the enrichment cycle cultures derived from the Florida Sun product, although *Saccharomyces* was the dominant genus, especially from the 2nd cycle on with more than 90% abundance. Further analysis of the metagenomic data of the enrichment cultures derived from the Florida Sun product identified four genera of bacteria, *Bacillus*, *Leuconostoc*, *Lactococcus*, and *Lactobacillus* in all of the enrichment cycle cultures, although *Lactobacillus* was the dominant genus, representing more than 95% of the abundance from the 2nd cycle onwards (Figure 4e).

### 3.3. Biocontrol Potential of Fresh and MA-Kefir Grains

**Figure 5:**
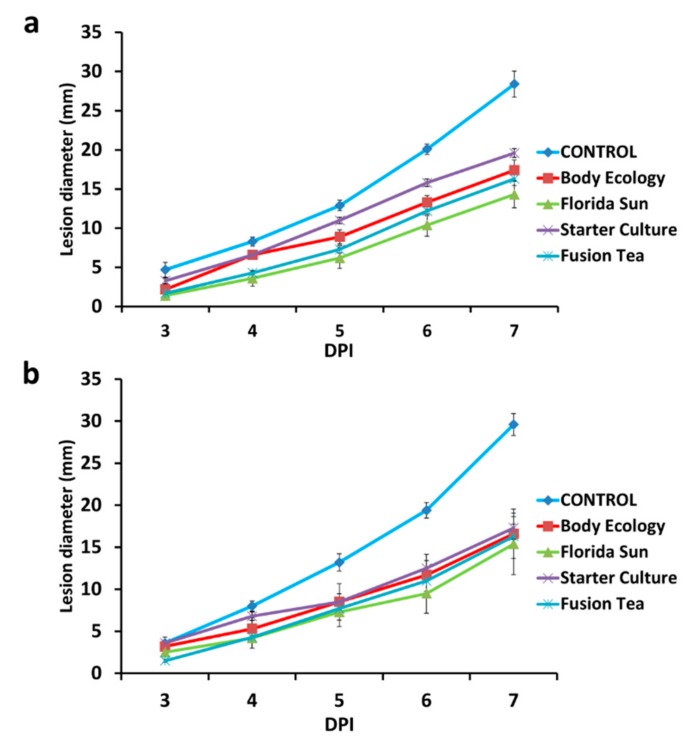
Effect of application of kefir grains as fresh (**a**) or milk-activated (**b**) on decay severity (lesion diameter of the wounds) on apple. Fruit were treated with suspensions of kefir grains and inoculated 2 h later with *Penicillium expansum* (10^5^ spores ml^−1^) or 20 μL sterile distilled water in control, and measurements were taken at 3, 4, 5, 6, and 7 days post inoculation (DPI). Bars indicate standard errors of the means.

All four of the kefir grain products (Fusion Tea, Starter Culture, Florida Sun, and Body Ecology) applied as fresh or MA-kefir at 1:10 dilutions of 1 g of grains homogenized in 1 mL of sterile distilled water reduced the lesion diameter in wounded apples inoculated with *P. expansum,* relative to lesion diameter in control wounds treated with water and then inoculated with the pathogen (Figure 5). Among the different products, microbial solutions obtained from the Florida Sun and Fusion Tea products exhibited the highest reduction in decay severity with up to a 50% reduction in lesion diameter compared to the control after 7 DPI. No significant effect was evident on decay incidence (percent of infected wounds) with either the fresh or MA-kefir grains, although fresh grains of the Florida Sun product exhibited a marginal reduction (20% and 30% after 3 and 4 DPI, respectively) in decay incidence, and MA-kefir grains exhibited a 20% reduction after 3 and 4 DPI and 10% at 5, 6 and 7 DPI.

### 3.4. Biocontrol Activity of Kefir Grains in Successive Enrichment Cultures

**Figure 6:**
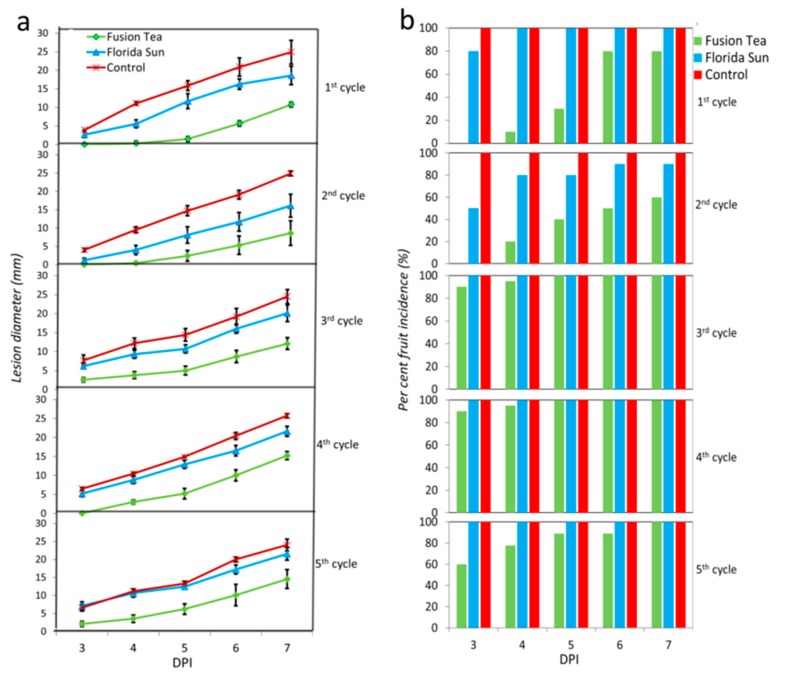
Biological activity of enrichment cultures of Florida Sun and Fusion Tea kefir grain samples (**a**—lesion diameter of the wounds; **b**—per cent wound infected). Fruit were treated with suspensions of kefir grains and inoculated 2 h later with *Penicillium expansum* (10^5^ spores ml^−1^) or 20 μL sterile distilled water in control and measurements were taken at 3, 4, 5, 6, and 7 days post inoculation (DPI). Bars indicate standard errors of the means.

The enrichment cultures from each of the five successive cycles of cultures derived from the Florida Sun and Fusion Tea kefir grains were assayed for biocontrol activity against *P. expansum* in apple wounds. The enrichment cultures derived from both products exhibited a small level of biocontrol activity, relative to the control (wounds treated with water and then inoculated with the pathogen) (Figure 6). Enrichment culture suspensions derived from the Fusion Tea product displayed the highest biocontrol activity after the 1st cycle, exhibiting a 20% reduction in disease incidence and a 50.6% reduction in lesion diameter, relative to control wounds. Biocontrol activity of the Fusion Tea product markedly increased at the 2nd cycle, exhibiting a 40.0% reduction in disease incidence and 65.5% reduction in lesion diameter at 7 DPI, relative to control wounds. Notably, the enrichment cultures derived from both the Fusion Tea and Florida Sun products exhibited higher biocontrol activity then treatments derived from either fresh or MA-kefir grains of the same products.

### 3.5. Biocontrol Activity of Individual Yeasts and Bacteria Isolated from Kefir Grains

**Figure 7:**
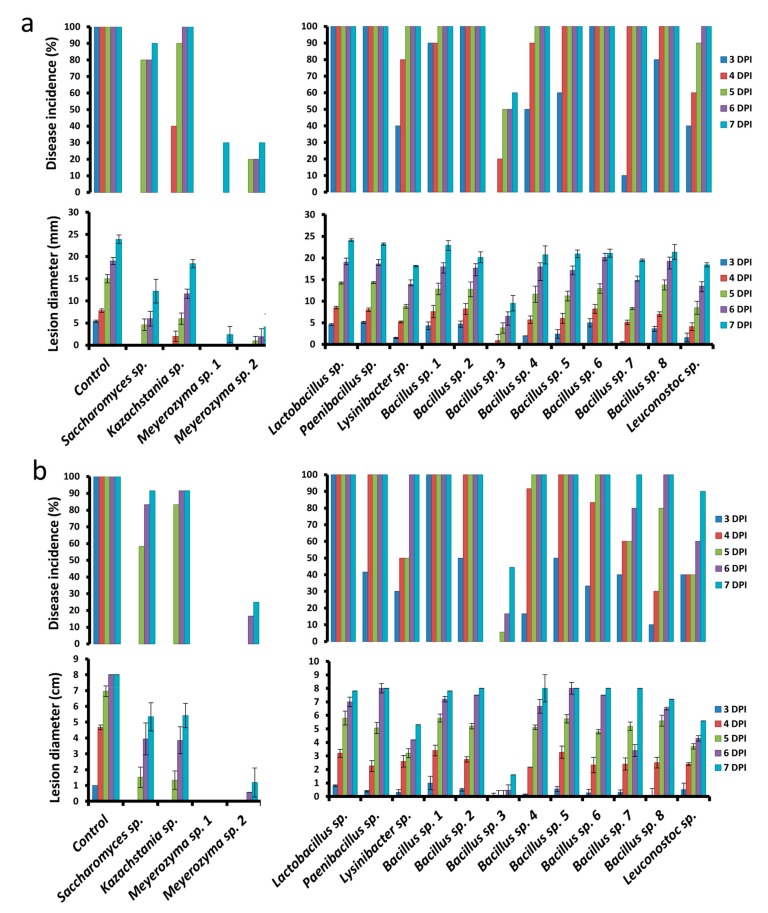
Effect of bacterial and yeast isolates from kefir grains on disease incidence (lesion diameters of the wounds) and severity (percent wounds infected) against *Penicillium expansum* on apple (**a**) and P. digitatum on grapefruit (**b**). Fruit were treated with suspensions of individual isolates (10^8^ CFU ml^−1^) and inoculated 2 h later with *P. expansum* or P. digitatum (10^5^ spores ml^−1^) or 30 μL sterile distilled water in control and measurements were taken at 3, 4, 5, 6, and 7 days post-inoculation (DPI). Bars indicate standard errors of the means.

Individual isolates (4 yeasts and 12 bacteria) isolated from the kefir grain products were selected after a preliminary investigation of 120 isolates (data not shown) and their potential biocontrol activity was assayed against *P. digitatum* and *P. expansum* on harvested “Star Ruby” grapefruit and “Granny Smith” apples, respectively. The individual isolates, applied to fruit wounds at a concentration of 10^8^ CFU ml^−1^, displayed varying degrees of reduction in disease incidence and severity against *P. digitatum* and *P. expansum* (applied at 10^5^ CFU ml^−1^) at different storage times (Figure 7a,b).

Among the yeast isolates, *Meyerozyma* sp. 1 was the most effective on grapefruit, exhibiting complete inhibition of the pathogen in inoculated wounds up to 7 DPI. This was followed by *Meyerozyma* sp. 2, exhibiting 75% reduction in disease incidence and 85.3% reduction in decay severity, relative to the control. Among the bacterial isolates, *Bacillus* sp. 3 was the most effective, exhibiting a 36.0% reduction in disease incidence and 80.0% reduction in decay severity, relative to the control, at 7 DPI.

*Meyerozyma* sp. 1 and 2 also exhibited the best biocontrol activity on apple against *P. expansum*, with each exhibiting a 70% reduction in disease incidence and 89.9% and 82.5% reduction in decay severity, respectively, relative to the control, at 7 DPI. Similarly, *Bacillus* sp. 3 exhibited the highest biocontrol activity among the various bacterial isolates, exhibiting a 40% reduction in disease incidence and a 61.2% reduction in decay severity, relative to the control.

## 4. Discussion

Kefir grains are complex biological entities comprising diverse species of microorganisms) that have been used for centuries to create fermented drink products from either milk or water. More recently, studies have shown that the microorganisms present in probiotic water kefir have a capacity to act biocontrol agents in stored grains [38]. Utilizing such consortia, or the organisms within them, for postharvest biocontrol, would represent a novel approach to providing both decay control and a source of probiotics. In the present study, the microbial analysis of the kefir grains revealed the coexistence of a stable population of a consortium of yeasts and bacteria, although their distribution and abundance varied between the kefir grains obtained from different commercial kefir-grain products. A higher population (CFUs) of yeasts than bacteria was observed across all the different sampling conditions of the kefir grains. Notably, however, species diversity of the bacteria isolated from the grains was much higher than the species diversity of yeasts. In the results obtained using a culture-dependent approach, *Bacillus* was the most common genus of bacteria out of the seven genera isolated, while, surprisingly, only four genera of yeasts could be identified from the kefir grains. Several studies have characterized the microbial diversity of kefir grains from different origins and sources and demonstrated that the microbial composition of kefir grains varies according to origin, substrate used in the fermentation process, and culture maintenance methods [39,40,41,42].

Metagenomic analysis of the kefir grains revealed that the overall bacterial community was dominated by *Lactobacillus*, *Lactococcus*, *Streptococcus,* and *Leuconostoc*, which corroborates the results obtained in previous studies of other kefir grain sources [43,44,45,46]. In addition to the large but variable bacterial diversity in kefir grains, there is also an abundant and diverse community of yeasts existing in a symbiotic relationship with the bacteria [40,44]. Our metagenomic analysis revealed the dominance of *Saccharomyces*, *Kluyveromyces,* and *Kazachstania* in all of the kefir grain products. In the current study, *Saccharomyces* was also the only common species isolated from all of the kefir grain products using a culture-dependent approach. The majority of previous studies have also reported *Saccharomyces*, *Kluyveromyces*, and *Candida* as the most commonly isolated yeasts from fresh or MA-kefir grains, and that these genera typically comprise the bulk of the total yeast population [39,42,44]. Notably, the cultured based method did not identify the majority of the organisms revealed by metagenome sequencing. This may be attributed to either their presence in extremely low numbers or their inability to grow on selected culture media, which cannot duplicate the complex conditions and interactions present in the kefir grains.

Homogenized solutions of kefir grains, either as fresh grains, MA-kefir grains, or as suspensions derived from enrichment cultures, exhibited biological control properties against the apple blue mold fungus, *P. expansum* in vivo. The enhanced biocontrol activity of the suspensions derived from the enrichment cultures of kefir grains as compared to either fresh or MA-kefir grains may be attributed to the presence of a higher initial microbial CFUs, both bacteria and yeasts, in the enriched cultures compared to fresh or MA-kefir grains. The consortia of microbes obtained through periodic enrichment of Florida Sun and Fusion Tea products exhibited promising biocontrol activities against *P. expansum* on apples, and metagenomic analysis of these consortia in each successive cycles revealed the presence of several yeast and bacterial genera growing together and contributing to the antagonistic activity.

Previous studies have indicated a range of biocontrol mechanisms may be operative in mixed populations of microorganisms, which may provide enhanced [47,48,49], reduced [50,51,52], or similar biocontrol efficacy [53,54,55]. Importantly, however, the relative significance of single and combinations of organisms in biocontrol activity was not established since testing for synergistic, enhanced, or antagonistic interactions among the microorganisms in controlling diseases was not conducted or statistically evaluated. Nevertheless, it is still apparent that the symbiotic community comprising several species of yeasts and bacteria that naturally occur in these kefir grains interacted and inhibited the growth of *P. expansum* in apple wounds to varying degrees in regards to disease incidence and severity.

Among the individual species of bacteria and yeasts examined for biocontrol activity, the two *Meyerozyma* sp. 1 and 2, *Saccharomyces* sp. and *Bacillus* sp. 3 were the most effective isolates. Notably, all of these species represent established biocontrol agents that are effective across a wide range of postharvest pathogens on several different commodities [56,57,58,59].

In conclusion, effective inhibition of *P. expansum* was observed with the use of the natural consortium of probiotic microbial species present in kefir grains. Several species of bacteria and yeasts were identified that exhibit biocontrol activity against *Penicillium* rots on apples and grapefruit. Further research needs to be conducted to determine if these probiotic species with antagonistic properties can be combined to generate a probiotic, ecofriendly, formulation with a broader efficacy against a wide range of postharvest pathogens on several horticultural commodities.
